# Medical Education Partnership Initiative (MEPI) in Zimbabwe: Outcomes and Challenges

**DOI:** 10.9745/GHSP-D-17-00052

**Published:** 2018-03-21

**Authors:** James G Hakim, Midion M Chidzonga, Margaret Z Borok, Kusum J Nathoo, Jonathan Matenga, Edward Havranek, Frances Cowan, Melanie Abas, Eva Aagaard, Susan Connors, Sanele Nkomani, Chiratidzo E Ndhlovu, Antony Matsika, Michele Barry, Thomas B Campbell

**Affiliations:** a Department of Medicine, University of Zimbabwe College of Health Sciences, Harare, Zimbabwe.; b Dean's Office, University of Zimbabwe College of Health Sciences, Harare, Zimbabwe.; c Department of Pediatrics and Child Health, University of Zimbabwe College of Health Sciences, Harare, Zimbabwe.; d Division of Cardiology, University of Colorado, Aurora, Denver, CO, USA.; e Research Department of Infection and Population Health, University College London, London, UK.; f Institute of Psychiatry, King's College London, London, UK.; g Division of General Internal Medicine, University of Colorado, Aurora, Denver, CO, USA.; h Evaluation Center, School of Education and Human Development, University of Colorado, Aurora, Denver, CO, USA.; i NECTAR Office, University of Zimbabwe College of Health Sciences, Harare, Zimbabwe.; j Department of Medicine, Stanford University, Palo Alto, CA, USA.; k Division of Infectious Diseases, University of Colorado, Aurora, Denver, CO, USA.

## Abstract

**Background:**

Sub-Saharan Africa has an inadequate number of health professionals, leading to a reduced capacity to respond to health challenges, including HIV/AIDS. From 2010 to 2015, the Medical Education Partnership Initiative (MEPI)—sponsored by the U.S. Presidents Emergency Plan for AIDS Relief (PEPFAR) and the National Institutes of Health (NIH)—was enthusiastically taken up by the University of Zimbabwe College of Health Sciences (UZCHS) and 12 other sub-Saharan African universities to develop models of training to improve medical education and research capacity. In this article, we describe the outcomes and challenges of MEPI in Zimbabwe.

**Methods:**

UZCHS in partnership with the University of Colorado, Denver; Stanford University; University of Cape Town; University College London; and King's College London designed the Novel Education Clinical Trainees and Researchers (NECTAR) program and 2 linked awards addressing cardiovascular disease and mental health to pursue MEPI objectives. A range of medical education and research capacity-focused programs were implemented, including faculty development, research support, mentored scholars, visiting professors, community-based education, information and technology support, cross-cutting curricula, and collaboration with partner universities and the ministries of health and education. We analyzed quantitative and qualitative data from several data sources, including annual surveys of faculty, students, and other stakeholders; workshop exit surveys; and key informant interviews with NECTAR administrators and leaders and the UZCHS dean.

**Findings:**

Improved Internet connectivity and electronic resource availability were early successes of NECTAR. Over the 5-year period, 69% (115 of 166) of faculty members attended at least 1 of 15 faculty development workshops. Forty-one faculty members underwent 1-year advanced faculty development training in medical education and leadership. Thirty-three mentored research scholars were trained under NECTAR, and 52 and 12 in cardiovascular and mental health programs, respectively. Twelve MEPI scholars had joined faculty by 2015. Full-time faculty grew by 36% (122 to 166), annual postgraduate and medical student enrollment increased by 61% (75 to 121) and 71% (123 to 210), respectively. To institutionalize and sustain MEPI innovations, the Research Support Center and the Department of Health Professions Education were established at UZCHS.

**Conclusion:**

MEPI has synergistically revitalized medical education, research capacity, and leadership at UZCHS. Investments in creating a new research center, health professions education department, and, programs have laid the foundation to help sustain faculty development and research capacity in the country.

## Abstract (precis)

The 5-year medical education and research strengthening initiative in Zimbabwe increased faculty retention and student enrollment, improved information technology infrastructure, provided mentoring for postgraduates and clinical training in specialty areas, instituted a competency-based curriculum reform process, and created new departments and centers to institutionalize health education and research implementation. A comprehensive review of the curriculum is still underway and uptake of technology-assisted teaching has been slower than expected.

## INTRODUCTION

The ongoing shortage of health workers in sub-Saharan Africa severely undermines the ability of countries to treat diseases and deliver essential health services. This health workforce crisis contributes to poor health outcomes, which are further compounded by the burden of HIV/AIDS.^1^ One solution to addressing this crisis is to strengthen medical education and increase capacity in research and clinical training to retain qualified medical professionals.

The trajectory of medical education in sub-Saharan Africa has been turbulent, with overall growth seen in the early independence years for many countries (1960–1975), followed by a period of deterioration fueled by political and economic instability (1975–1990).^4^ The number of medical schools increased sharply from 54 in 1980 to 168 in 2009. Despite an increase in the number of doctors trained, there is still a severe shortage of medical practitioners in Africa, largely because of a “brain drain”—the flight of skilled medical professionals to other countries.^3^

The trajectory of medical education in sub-Saharan Africa has been turbulent.

In Zimbabwe specifically, efforts to strengthen medical education were severely hampered by the economic and political crisis from 1999 to 2009, which resulted in a 70% reduction in medical student enrollment, 61% faculty vacancy rate, and a near collapse of specialist training by 2010.^3^ The University of Zimbabwe College of Health Sciences (UZCHS), the only medical school in Zimbabwe until 2010, faced declines in infrastructure and limited capacity to use technological innovations in education and research.^5^ UZCHS was trapped in a vicious cycle, with the emigration of existing faculty and an inability to attract candidates for specialist training, leading to low faculty-to-student ratios, and in turn, low enrollment and graduation of students.

The University of Zimbabwe College of Health Sciences was trapped in a vicious cycle, leading to low enrollment and graduation of students.

These challenges were mirrored by national shortages in the overall health workforce, with a ratio of 142 health workers per 100,000 people in 2009, which is far below the World Health Organization minimum threshold of 230 health workers per 100,000 people required to deliver essential health services.^6^ In addition, Zimbabwe faced a sharp demand to expand health services to address the HIV/AIDS epidemic, which escalated sharply in the 1980s and 1990s. Disparities in health service provision between urban and rural areas was also a critical problem because the majority (70%) of the Zimbabwean population is rural.^7^

To address this problem in Zimbabwe and related problems in other sub-Saharan African countries, the U.S. President's Emergency Plan for AIDS Relief (PEPFAR), with financial support from the National Institutes of Health (NIH), launched the Medical Education Partnership Initiative (MEPI) in sub-Saharan Africa in 2010 to implement robust medical education models and improve research capacity in HIV/AIDS, non-communicable diseases, and other health priorities.^2^ The 3 core themes of MEPI are to (1) increase capacity by enhancing the quantity and quality of medical education, (2) retain faculty and graduates in order to strengthen the capacity of schools and graduates in their respective countries, and (3) ensure regionally relevant research to generate new knowledge and to strengthen and retain faculty.^2^ MEPI grants were awarded to 13 medical schools in 12 sub-Saharan African countries— Botswana, Ethiopia, Ghana, Kenya, Malawi, Mozambique, Nigeria, South Africa (2 schools), Tanzania, Uganda, Zambia, and Zimbabwe.^2^

In this article, we provide a detailed description of how MEPI was implemented in Zimbabwe—specifically, the Novel Education Clinical Trainees and Researchers (NECTAR) program and 2 smaller linked programs pursuing cardiovascular and mental health research—and explore the outcomes and challenges of the initiative in the country.

## MEPI IMPLEMENTATION IN ZIMBABWE

A critical core of faculty at UZCHS continues to be dedicated to the advancement of medical education and research despite historical challenges. In 2010, they received a MEPI award of US$13 million to improve medical education and to strengthen medical research and clinical training. MEPI activities were implemented through faculty development, research support, mentored scholars, visiting professors, community-based education, information and communication technology, cross-cutting curricula, and collaboration with partner universities and the ministries of health and education (Figure).

**FIGURE:**
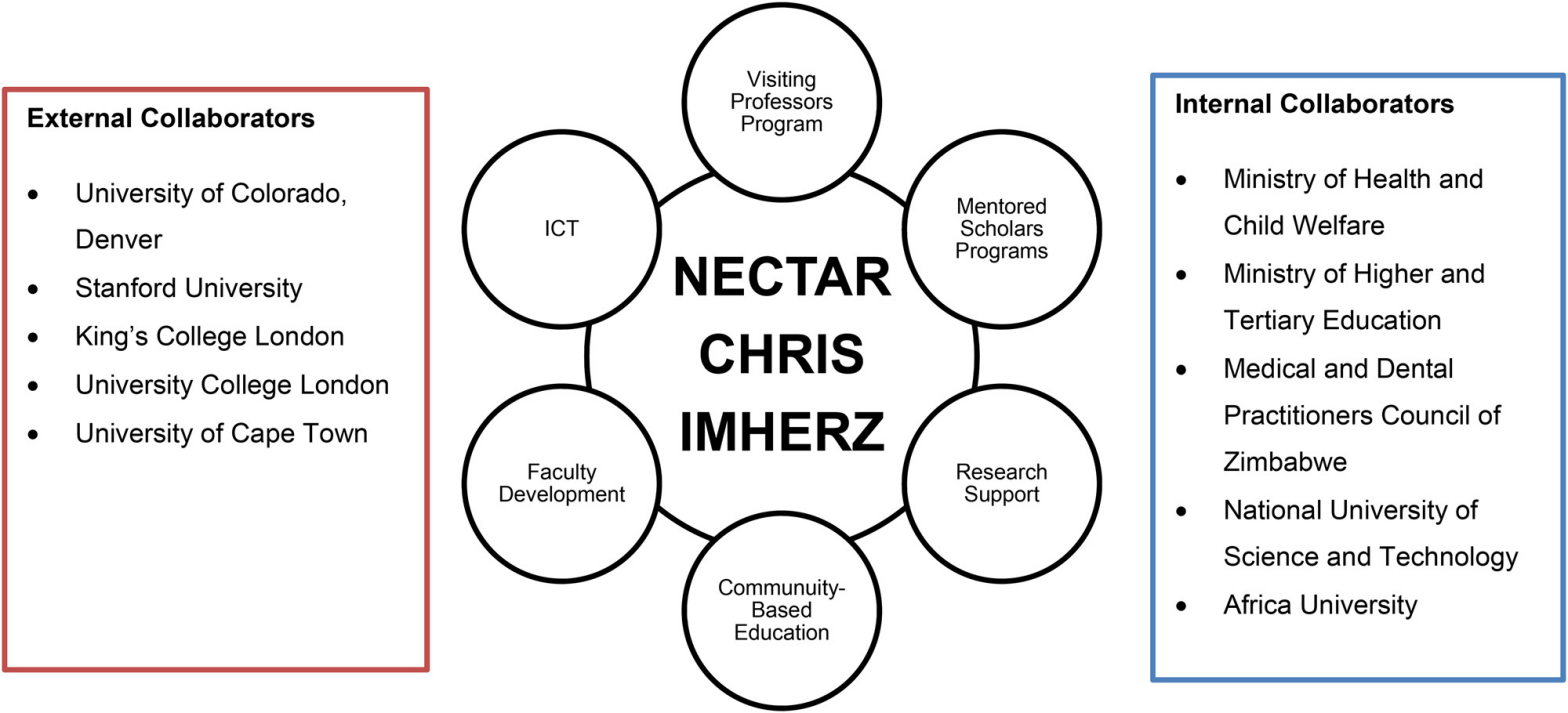
MEPI Programs and Collaborators in Zimbabwe

Abbreviations: CHRIS, Cerebrovascular Heart failure, Rheumatic heart disease Interventions Strategy; ICT, Information and Communication Technology; IMHERZ, Improving Mental Health Education and Research Capacity in Zimbabwe; NECTAR, Novel Education Clinical Trainees and Researchers.

NECTAR was a 5-year program established in 2010 by UZCHS in partnership with the University of Colorado, Denver (CU Denver) and Stanford University. Oversight and administrative support was provided by NIH, the U.S. Health Resources and Services Administration, Office of the U.S. Global AIDS Coordinator and Health Diplomacy, and the MEPI Coordinating Center.

UZCHS hired staff to form a MEPI secretariat to oversee the implementation of programs in close collaboration with the UZCHS dean's office. UZCHS faculty and administration were enthusiastic supporters of the programs following comprehensive sensitization and advocacy strategy efforts.

A major component of the programs was workshops facilitated by local and partner universities. UZCHS relied heavily on expertise from partner institutions at first, but the responsibility for facilitation gradually transitioned to local UZCHS staff after comprehensive training, capacity development, and mentorship. Toward the end of the 5-year MEPI grant, only a few highly specialized areas required input from partners.

### Faculty Development

Faculty development workshops were offered 3 times annually for all UZCHS faculty. In addition, an advanced 12-month course called the Health Education Advanced Leaders program for Zimbabwe (HEALZ) was designed to equip faculty with the necessary knowledge, skills, and attitudes to implement best practices in pedagogy, reliable learner assessment tools, and curriculum evaluation while building leadership and change-management skills. The cross-cutting academic subcommittee undertook curriculum development, particularly in PEPFAR priority areas—HIV, tuberculosis, and malaria.

### Research Support

NECTAR and grants from the Wellcome Trust and Netherlands government contributed to the establishment of infrastructure to support research policy development, skills building, and pre- and post-award grant implementation was an important aspect of research capacity strengthening. The construction of the Research Support Center was funded by this consortium, with the aim of institutionalizing research training (i.e., courses and workshops), disseminating information on funding and fellowship opportunities, and providing research administration. Training of research administrators and managers was also included in research capacity development.

### Mentored Scholars Programs

The Mentored Clinical Scholars Program and the Mentored Research Scholars Program sought to retain Master of Medicine (MMed) residents to serve as medical educators, investigators, and mentors at UZCHS. The Mentored Clinical Scholars Program offered training in medical education, leadership, and other skills through interactive workshops. The Mentored Research Scholars Program provided research training and support through workshops, administrative and financial assistance for research projects, and triangular mentoring (each scholar assigned to local and partner university mentors).

### Visiting Professors Program

The Visiting Professors Program was developed to enable experts from NECTAR partner universities to spend 2 to 4 weeks in Zimbabwe providing lectures, bedside tutorials, and other skills. This program was a key stop-gap measure that ensured that NECTAR and the linked-awards were able to launch its activities from the beginning of the grant. Through the program, 106 experts made 211 visits to UZCHS. The contribution made by visiting professors was critical in addressing the lack of expertise in several subspecialty areas. UZCHS MMed residents also made visits to partner universities for training and observation in specialized areas.

### Community-Based Education

Medical students spent between 2 and 4 weeks every year at district hospitals and other rural locations during their medical school training. This practice began in the 1980s to familiarize students with health determinants at non-urban and primary care settings.^8^ The curriculum, however, had not been revised since then. NECTAR provided improvements in Internet connectivity, housing infrastructure, and skills development at demonstration sites (Murewa and Howard district hospitals).^9^

### Information and Communication Technology

MEPI interventions were underpinned by investments in information and communication technology, which included expansion of college-wide availability of Wi-Fi, the installation of high-capacity data storage arrays, bandwidth increases, e-learning resources, and virtual private networks to facilitate information sharing between disparate clinical and teaching sites.

### Cardiovascular and Mental Health Research Programs

Two smaller linked awards—Cerebrovascular Heart failure, Rheumatic heart disease Interventions Strategy (CHRIS) and Improving Mental Health Education and Research Capacity in Zimbabwe (IMHERZ)—were designed to address research capacity and implementation needs in cardiovascular medicine and mental health, respectively. CHRIS and IMHERZ were developed in partnership with CU Denver, University of Cape Town, University College London, and King's College London.^3^

The CHRIS program aimed to enhance the cardiovascular curriculum, student and academic staff research capacity, and foster scholarship in cardiovascular disease knowledge and skills. This included theoretical and practical skills training in cardiology and neurology. The program later extended to training in endocrinology and pulmonology.

The IMHERZ scholars were trained in child psychiatry, forensic psychiatry, neuropsychiatry, and community psychiatry. Additionally, IMHERZ supported curriculum development, research skills development, and multidisciplinary master classes to strengthen capacity in certain areas (psychotherapy, autism, cognitive behavioral therapy, and substance abuse).

### MEPI Network

MEPI network activities were separate from the implementation of MEPI activities at the schools but were complementary and enabled adoption of best practices from the entire network. For example, 8 technical working groups were developed to provide a platform for all MEPI schools to pursue common academic and research interests in a community of practice model. The technical working groups focused on competency-based education, medical education research, community-based education, e-learning, research administration support, graduate tracking, monitoring and evaluation, and library and information science. Members met via Skype, email, webinars, workshops, and satellite meetings during MEPI symposia. Other MEPI network-wide programs that benefited the schools included the annual MEPI symposia, regular webinars, newsletters, and annual sponsor-led evaluation site meetings that included cross-site MEPI representation.

### Collaborations

In-country collaborators included the Ministry of Health and Child Care, the Ministry of Higher and Tertiary Education, professional councils, and other institutions. Internationally, UZCHS has longstanding collaborations with CU Denver, Stanford University, University College London, King's College London, and University of Cape Town. These institutions played key roles in the implementation and evaluation of NECTAR activities. The MEPI Principal Investigators Council, composed of principal investigators from the 13 MEPI beneficiary schools, was formed in 2011 to engender African leadership of MEPI activities. The council was instrumental in networking activities such as annual site visits and symposia. The council also oversaw the operations of the 8 technical working groups.^10^

## METHODS

We analyzed quantitative and qualitative data from several data sources available during the implementation of the NECTAR program. An evaluation team developed a comprehensive evaluation framework and implemented it in partnership with the Evaluation Center of CU Denver. The UZCHS-NECTAR evaluation team provided program leaders with regular and timely formative assessments to inform ongoing program improvement and summative assessments to monitor progress toward MEPI goals. The evaluation was guided by 3 questions: (1) How can the number of medical students completing training at UZCHS increase? (2) How prepared are graduates to remain in Zimbabwe to practice, conduct research, and teach future generations of doctors? (3) How can researchers strengthen their capacity to address the priority health care needs of the region? These questions align with the 3 MEPI thematic areas to improve medical education capacity (quality and quantity), promote the retention of health professionals, and conduct regionally relevant research.

This article draws on quantitative and qualitative data from the following sources:

- **Annual surveys (2011–2015, 2017).** Surveys were administered to faculty, undergraduate students (all disciplines) and postgraduate students. Data were collected on all programs that addressed the MEPI thematic areas. The 2017 survey was administered to faculty only to assess the extent to which faculty were using the teaching approaches introduced during the implementation of NECTAR. The Table shows the survey response rates of faculty, MMed residents, and undergraduate students.
- **MEPI annual institutional surveys (2011–2015).** The MEPI Coordinating Center administered these surveys to gather information on MEPI thematic areas. Data was gathered on student enrolment, attrition/pass rates and on faculty numbers and retention.
- **Workshop and training session exit surveys (2010–2015).** Exit surveys following workshops and training sessions provided feedback on whether expectations were met, what knowledge and skills were gained, and levels of satisfaction with the content.
- **Community-based education report (2014).** The data for this report were gathered from UZCHS faculty and administrative staff and from field training sites. It provided information on the strengths and weaknesses of UZCHS community-based education and made recommendations for improvement.
- **MEPI Network 5-year report (2015).** This was a compilation of reports from each of the 13 MEPI schools. It provided information on MEPI thematic areas, HIV/AIDS and program sustainability, functions of technical working groups, and the MEPI Principal Investigators Council.
- **NECTAR key informant interviews (2017).** These interviews explored the performance of the UZCHS Research Support Centre and the Department of Health Professions Education. We interviewed the directors and program officers of these units.
- **Other sources.** We also reviewed other sources of information including, report from annual site visits, and photo essays. MEPI sponsors organized annual site visits assisted by the MEPI coordinating center. The team gets updates on all NECTAR and linked award activities during a 3–4 day visit. They meet undergraduate and postgraduate students, faculty, the dean, Vice Chancellor and Ministry of Health and Education officials. The MEPI sponsors then makes a report available to the site documenting the visit. The photo essays were developed around faculty members identified by their peers to have made significant impact and shown leadership through their participation in NECTAR and the linked awards. There were 18 such champions.

**TABLE.** Survey Response Rates of Faculty, Master of Medicine Residents, and Undergraduates, 2011–2017

Respondents^a^	2011	2012	2013	2014	2015	2017
Faculty						
No. responded	28	68	55	49	43	34
No. approached	116	151	185	189	166	65
Percentage response rate	24%	45%	30%	26%	26%	52%
Master of Medicine residents						
No. responded	0	15	16	29	39	NA
No. approached	0	40	171	182	169	
Percentage response rate	0%	38%	9%	16%	23%	
Undergraduates						
No. responded	286	0	506	383	408	NA
No. approached	572	117	584	710	680	
Percentage response rate	50%	N/A	87%	54%	60%	

a Responses were collected during annual surveys from 2011 to 2015. A follow-up survey was conducted in 2017 among faculty to assess whether they were using the various teaching approaches introduced in the Novel Education Clinical Trainees and Researchers program.

**Figure:**
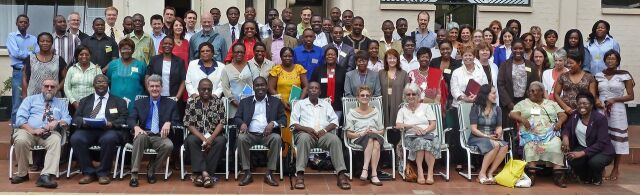
First annual MEPI partners' retreat in Harare, Zimbabwe, December 2011. © 2011 Ronald Nongwani

## FINDINGS

### Short-Term Programmatic Outputs

#### Faculty Development

Fifteen core faculty development workshops were conducted in 5 years, and 69% (115/166) of faculty members attended 1 or more workshops. The faculty development committee consisted of UZCHS and CU Denver members who selected workshop topics that included curriculum development, learner assessment methods, and adult teaching principles. In the 2015 annual faculty survey, 76% (33/43) of respondents felt they had gained new knowledge from participation in the workshops, 74% (32/43) felt they had gained new teaching skills, and 100% intended to incorporate their new knowledge and skills into teaching practice. Team-based learning and student assessment methods were most frequently cited as strategies incorporated into teaching practice. Medical students perceived an improvement in the quality of instruction over time, with clear objectives for each lecture, better prepared slides, and improved clinical teaching. In a follow-up survey in 2017, faculty respondents had implemented 1 or more key instructional innovations featured in the workshops, such as developing learning objectives, incorporating small-group work, or using single best answer multiple choice assessments.

By 2017, 5 cohorts of scholars (n=65) had completed the HEALZ program, with 3 cohorts (n=41) completing it during the grant period. As evidence of their value, 14 HEALZ scholars were nominated as members of the 24-member college-wide curriculum review committee. A survey performed among the first 3 HEALZ cohorts showed that 87% were highly satisfied with the training and perceived a positive change in knowledge on key content areas, such as curriculum development, analyzing quantitative and qualitative data, assessing learners, and writing for publication.

15 core faculty development workshops were conducted in 5 years, and 41 members of faculty had completed advanced training by 2015.

#### Curriculum and Other Learning Improvements

A new 18-month HIV/AIDS curriculum for MMed residents was developed and launched in 2011. In addition shorter tuberculosis and malaria curricula were developed for both MMed residents and undergraduate students. Between 2011 and 2015, 525 undergraduate medical students in their final year completed a 10-week HIV/AIDS course consisting of 10 sessions, delivered through a modified team-based learning approach. In a survey that included 120 students, 94% agreed and 71% strongly agreed that team-based learning was a more stimulating approach to learning compared with didactic lectures, and 96% felt that team-based learning enhanced their knowledge of HIV management.^5^

Through NECTAR, UZCHS initiated a competency-based curriculum review adapting the 7 patient-centered competency domains of the 2005 CanMEDS framework: medical expert, scholar/researcher, educator, community health advocate, communicator/relationship builder, manager/leader, and ethical/professional.^11^ In 2015, 79% of faculty respondents reported that they had begun the process of curriculum review. In 2017, 50% (17/34) reported their departments had completed the curriculum review and had developed a new working draft that was being implemented.

UZCHS initiated a competency-based curriculum review and by 2017, more than half of faculty respondents were in the process of implementing a new curriculum.

#### Enhanced Community-Based Education

In efforts to modernize its community-based education approach, UZCHS participated in a peer-review process through the Collaboration in Health Equity through Education and Research consortium.^9^ This review was supported by CapacityPlus, a program funded by the U.S. Agency for International Development that focuses on health workforce enhancement to achieve development goals. Peer reviewers included faculty from the universities of Botswana, Malawi, South Africa, and Zambia. The review provided useful feedback on the strengths and challenges of community-based education at UZCHS and suggested areas for improvement, such as strengthening coordination and conducting field programs in interdisciplinary teams.^9^ Other recommendations included modernizing the field attachment program to reflect new thinking on social determinants of health. Reviewers also recommended aligning the community-based education program with the overall medical school curriculum in UZCHS's ongoing exercise of curriculum review.

#### Mentored Scholars Programs

The Mentored Clinical Scholars Program conducted 11 workshops facilitated by UZCHS faculty and partners from CU Denver. Average attendance of MMed residents increased from 44 (31.4%) in 2013 to 86 (49.7%) in 2015. All UZCHS departments were represented at the workshop sessions. According to interviews of 10 frequent attendees and 2 infrequent attendees (n=12) in 2014, the majority felt the program filled a gap in preparation for a career in medical education. According to 7 of these attendees, the most beneficial topics were end-of-life care, death and dying, delivering bad news, and bereavement.

Participants felt that the Mentored Clinical Scholars Program filled a gap in preparation for a career in medical education.

The Mentored Research Scholars Program selected 33 scholars in 4 cohorts. In the final 2015 exit survey, 100% of surveyed scholars felt that they had engaged in relevant research, and 71% were highly satisfied with the mentoring experience. The vast majority (85%) reported they were more likely to pursue a career in research. By 2015, these scholars had contributed to research output through 7 international and national conference presentations, 4 accepted abstracts, 7 publications, and a noteworthy US$100,000 grant in reproductive, maternal, newborn, and child health awarded to a scholar.

#### Improved Research Support

Most faculty members (85.1%) and more than half (55%) of students were satisfied with the services provided by the Research Support Center. Both students and faculty members most frequently cited the research training workshops as the most used service. The majority (79%) of faculty members thought their research skills had been enhanced through the Research Support Center. In 2017, 46% (16/34) of faculty respondents who participated in research training and used the Research Support Center reported they had made great progress in their medical research during the previous year.

#### Technology and Library Services

The NECTAR program established Wi-Fi connectivity at the college and major teaching sites, which was an important milestone given the previously limited connectivity. A range of online research and learning support resources were also made available such as Research Electronic Data Capture (REDCap),^12^ eGranary Digital Library,^13^ Hinari,^14^ UpToDate, and Stanford University course materials.^15^ More than 400 students and 76 academic staff received training in online research and training support resources and learning management systems.

In 2015, 85% of faculty members surveyed were satisfied with the improvements in access to technology since the beginning of NECTAR, compared with 51% of MMed residents and 44% of medical students who felt satisfied. All (100%) faculty surveyed (n=43) and 72% (n=39) of MMed residents rated the online research and learning support resources as very useful; however, 47% (n=190) of medical students had insufficient knowledge of the resources to rate their usefulness.

#### HIV/AIDS Care

NECTAR was conceived to address the lack of a competent and responsive health workforce to deliver HIV services, especially antiretroviral therapy. In 2011, NECTAR developed and immediately implemented HIV/AIDS curricula for medical students and MMed residents in all disciplines. The benefits of this HIV/AIDS curriculum were noticed in the main teaching hospitals first, and then extended out as doctors and specialists became more qualified and spread to other parts of the country.

NECTAR implemented new HIV/AIDS curricula for medical students and MMed residents, the benefits of which were first seen in teaching hospitals and then other parts of the country.

#### Strengthening Cardiovascular Expertise and Research Capacity

The CHRIS program offered cardiovascular training to 52 scholars in 4 cohorts: 17 MMed residents, 9 sonographers in echocardiography, 5 junior academic staff, and 21 physiology intercalated students (competitively selected second-year medical students who spent 1 year working for research degrees in physiology). CHRIS scholars were trained in a range of cardiology-related skills, including electrocardiogram interpretation, stress testing and ambulatory recording, pacemaker implantation, pediatric cardiology, and Holter monitoring. In neurology, the specialist management of stroke patients and interpretation of electroencephalograms for optimal management of epilepsy patients were key areas of training. In addition, respiratory services were expanded with capacity to do bronchoscopy and spirometry. Disease registries were established to generate local evidence to support the establishment of a stroke unit and adult cardiac research laboratory, and 5 new specialist clinics (neurology/stroke, cardiology, diabetes/endocrinology, pediatric cardiology, and epilepsy).

Scholars were trained in a range of specialized cardiology and neurology skills, and new disease registries led to the establishment of a stroke unit and adult cardiac research laboratory, and 5 specialist clinics.

#### Strengthening Mental Health Expertise and Research Capacity

Through the IMHERZ program, 6 Masters degrees and 15 diplomas in psychiatry were awarded between 2010 and 2015. The program included 10 master classes for nurses, clinical psychologists, occupational therapists, and social workers on various topics—HIV adherence, autism, cognitive behavioral therapy, and substance abuse. Feedback from the master classes demonstrated that participants had increased confidence in managing mental health problems. IMHERZ gave rise to new curricula in behavioral science for preclinical students and 2 undergraduate modules on mood disorders and depression. The program established 4 community outreach clinics, which are considered pilot projects for nationwide replication. IMHERZ scholars also facilitated the establishment of child psychiatry clinics at the 2 central hospitals in Harare.

### Medium-Term Programmatic Outcomes

#### Addressing Faculty and Student Number Deficits and Retention

The long-term impact of MEPI in Zimbabwe has yet to be realized, a number of achievements are notable. Between 2010 and 2015, full-time academic staff numbers at UZCHS grew by 36% (from 122 to 166 posts), annual postgraduate enrollments increased by 61% (75 to 121), and annual medical student intakes rose by 71% (from 123 to 210). Twelve MEPI scholars have already joined faculty as junior lecturers. Survey results show that the number of medical students who intend to practice in Zimbabwe after graduation grew by 9% between 2011 and 2015, and more students felt that staying in Zimbabwe would enable them to get a good job, earn a living wage, do work that is satisfying, and earn respect from others in their field. The department of psychiatry experienced a fivefold increase in staff from just 1 member in 2010 to 5 in 2015. All 21 intercalated graduates in physiology and anatomy were engaged as teaching assistants and tutors. The number of medical practitioners registered to practice in Zimbabwe increased by 32.6% between 2011 and 2014 (from 2,003 to 2,656). This increase was due to a combination of factors including the return of medical practitioners who had left the country.

Between 2010 and 2015, full-time academic staff numbers at UZCHS grew by 36%, annual postgraduate enrollments increased by 61%, and annual medical student intakes rose by 70%.

After MEPI ended in 2015, faculty retention and student enrollment rates remained high. Of the faculty who were recruited during MEPI, 97% (58 of 60) were still at UZCHS in 2017. Annual enrollment of MMed residents increased by 12.5% (from 48 to 54) from 2015 to 2017 and the enrollment of medical students increased by 10.5% (from 210 to 232) during the same period. NECTAR enabled higher student graduation rates and better retention of graduates and faculty; however, improvements in the socioeconomic environment were also a key contributor.

After MEPI ended in 2015, faculty retention and student enrollment rates remained high.

#### Department of Health Professions Education

The Department of Health Professions Education was established in the third year of the NECTAR program to streamline professional health education at UZCHS. The establishment of this department was a crucial step in institutionalizing NECTAR activities and innovations. The core function of the department is to provide teaching and learning support and to oversee curriculum development and implementation. After the MEPI grant period, the department continued to organize 2 faculty development workshops per year with funding from the University of Zimbabwe. In 2015, a total of 157 faculty members were trained—114 in 2016 and 34 in early 2017. All these faculty members attended at least 1 workshop.

Under the HEALZ advanced faculty development program, 3 cohorts were trained—41 in 2015, 9 in 2016, and 15 in 2017. In the 2017 follow-up survey, 97% (33/34) of respondents agreed or strongly agreed that UZCHS maintained its MEPI goal to improve the quality of health education.

#### Research Support Center

The Research Support Center has supported faculty in research output, evidenced by 31 publications, 86 abstracts, and 37 presentations in local, regional, and international conferences from 2010 to 2015. The Research Support Center assisted in the submission of 23 successful grant applications worth more than US$16 million. In addition, the center has played a critical role in training researchers and providing administrative support. In 2014, more than half (55%, n=29) of MMed residents surveyed were satisfied with services provided and the majority (77%, n=29) felt their research skills were enhanced.

The Research Support Center has supported faculty in research output, evidenced by 31 publications, 86 abstracts, and 37 presentations from 2010 to 2015.

Since the MEPI grant ended, the Research Support Center has increasingly supported itself financially by charging overhead on grants and courses. In 2016, it supported 29 applications (8 of which were successful) and in 2017, it supported 9 applications (2 of which were successful by midyear). The number of grants managed by the center increased from 15 in 2015 to 21 in 2017.

#### Future Collaborations

MEPI partner universities are committed to working with UZCHS to ensure the sustainability of NECTAR innovations. The Department of Medicine at CU Denver has provided ongoing support for the Visiting Professors Program through a faculty and residents exchange program, the Colorado Zimbabwe International Exchange. Stanford University supports a faculty and residents exchange program to address the gaps in the disciplines of surgery, otorhinolaryngology, and anesthesia, in addition to the Global Health Equity Scholars Fellowship. Structured mentored research opportunities will continue to be available to junior faculty through 2 new programmatic grants—Promoting Excellence in Research and Faculty Enhanced Career Training (PERFECT) funded by NIH ^16^ and the African Mental Health Research Initiative funded by the Wellcome Trust.^17^ In the last year of the MEPI grant, the Principal Investigators Council was actively engaged in developing an expanded African continent-wide forum (including nurses, midwives, and other health cadres) to continue to foster the transformative gains and best practices learned through MEPI.

#### Medical Subspecialist Services

Data have shown that establishment of the stroke unit has reduced first-week stroke-related mortality from 24.7% to 13.7% due to the introduction of interventions to reduce inhalation and consequent aspiration pneumonia. By 2017, the 8-bed stroke unit had admitted more than 1,000 patients since it was established in 2013. The Ministry of Health and Child Care has recognized the impact of the stroke unit and is planning to replicate the model in hospitals countrywide.

The Ministry of Health and Child Care is planning to replicate the stroke unit model in hospitals countrywide.

The subspecialty training in mental health through IMHERZ has made a substantial impact on the improvement of services in several sectors in the country. For example, with the availability of psychiatrists trained in forensic psychiatry, forensic services are now available to help inmates after a long stint without these services. IMHERZ was also a key player in drafting the mental health policy of Zimbabwe, which is now a key Ministry of Health document.

#### Implementation Challenges and Supporting Factors

In 2012, faculty reported some challenges in implementing MEPI activities during the first 2 years. The primary challenge noted was finding protected time for active participation because of the multiple educational and clinical demands and responsibilities of UZCHS faculty and leaders. Given clinician and instructor shortages, these challenges were not completely addressed during the grant period; however, in later years, some compensation was provided for faculty who participated in extended trainings. Faculty also described the challenges related to communication across programs, departments, and institutions in the early years. In 2014, faculty indicated that effective communication mechanisms evolved over time including establishing regular meeting times and distributing meeting agendas and minutes.

Factors that contributed most to the programs' progress and success were effective leadership, a high degree of investment of faculty and ministry leaders, and the rapid establishment of the program infrastructure and visibility, according to a survey among faculty in 2012. In 2014, faculty members reported that the intentional involvement of all UZCHS departments and key faculty leaders was an effective strategy in moving toward achieving program goals.

Successful program factors included effective leadership, investment of faculty and ministry leaders, and the rapid establishment of infrastructure and visibility.

##### Sustainability of MEPI

Immediately after MEPI was awarded in 2010, the NECTAR team engaged the University of Zimbabwe vice chancellor and other key stakeholders to share the purpose of the program, its implementation plan, and the necessary preparations to sustain these activities after the MEPI award period. During NECTAR, the university leadership was regularly informed and invited to participate and officiate at all key program activities. This resulted in a great appreciation of the program and strong buy-in among all stakeholders. The Department of Health Professions Education and the Research Support Center were given university departmental status and were allocated funds. As mentioned earlier, the Research Support Center is increasingly improving its income base by charging overhead costs.

Improvements in information and communication technology services such as Wi-Fi, e-resources, high capacity storage, and data management systems have continued after the MEPI grant. The challenge is the capacity to maintain and upgrade these once the need arises.

The collaborating external universities have taken over funding of the visiting professors and resident exchange program. Further awards have continued to promote capacity development in research at UZCHS.

## CONCLUSION

MEPI has brought about a renaissance in medical education at institutions in sub-Saharan Africa. Given the short life span of MEPI grants, however, sustainability has been a challenge at MEPI schools. NECTAR emphasized strengthening local capacity to sustain programs beyond the grant, and investments in infrastructure such as the Department of Health Professions Education and Research Support Center, are cornerstones for the sustainability of faculty development and research capacity. The Mentored Clinical Scholars Program and Mentored Research Scholars Program for postgraduates have created a pipeline of future academics with the capacity to build on gains made in the past 5 years. Furthermore, annual surveys revealed increased commitment from faculty members to sustain and expand on achievements made during the MEPI grant period.

MEPI has brought about a renaissance in medical education at institutions in sub-Saharan Africa.

A comprehensive review of the curriculum and the community-based education program will take longer to accomplish; however, NECTAR has laid the necessary foundation to achieve this goal. The uptake of technology-assisted learning and teaching has been slower than expected despite improvements in infrastructure, but new programs are committed to continuing progress in this area. The Department of Health Professions Education is focused on improving online research and learning support resources at UZCHS as a special focus area, buoyed by the overall commitment to accelerated technology by the university. Notable improvements in retention of faculty have been made; however, retention of medical practitioners is likely to remain a challenge in Zimbabwe because of many factors that are beyond the scope of NECTAR and UZCHS.

## Notes

**Peer Reviewed**

**Funding:** PEPFAR, NIH.
